# Resistin levels and inflammatory and endothelial dysfunction markers in obese postmenopausal women with type 2 diabetes mellitus

**DOI:** 10.1186/s13098-021-00715-7

**Published:** 2021-09-08

**Authors:** A. Giandalia, A. Alibrandi, L. Giorgianni, F. Lo Piano, F. Consolo, G. Longo Elia, B. Asztalos, D. Cucinotta, G. Squadrito, G. T. Russo

**Affiliations:** 1 Department of Clinical and Experimental Medicine, University of Messina, Messina, Italy; 2 Unit of Statistical and Mathematical Sciences, Department of Economics, University of Messina, Messina, Italy; 3 Lipid Metabolism Laboratory, JM-USDA-Human Nutrition Research Center on Aging at Tufts University, Boston, MA USA

**Keywords:** Resistin, Inflammation, Women, Type 2 diabetes, CHD

## Abstract

**Background:**

Obesity-associated coronary heart disease (CHD) risk is higher in women than in men with type 2 diabetes (T2DM). Resistin, an adipokine secreted by adispose tissue, may contribute to this higher risk.

**Aims:**

To explore the relationships among resistin levels and common inflammatory and endothelial dysfunction markers and CHD risk in obese post-menopausal T2DM women.

**Methods:**

Serum levels of resistin, hsCRP, IL-6, Soluble vascular cell adhesion molecule (sVCAM), homocysteine (tHcy), HOMA-IR and metabolic parameters were determined in a group of 132 T2DM women with and without documented CHD and in 55 non-diabetic women.

**Results:**

Resistin, sVCAM, IL-6 and tHcy levels were comparable in T2DM and controls. CHD women showed higher resistin, sVCAM and tHcy levels than those without CHD, and for resistin this difference remained significant after age-adjustment (P = 0.013); conversely hsCRP were ~ 2X higher in T2DM women than in controls (P = 0.0132) without any difference according to CHD history. At univariate analysis resistin levels were significantly associated with age, waist circumference, hypertension, tHcy, hsPCR, sVCAM, IL-6, HDL-cholesterol, triglycerides and creatinine levels, but only creatinine, triglycerides, hsCRP, IL-6 and sVCAM were independently associated to resistin levels at stepwise regression analysis.

Resistin levels were independently associated to CHD, increasing the risk by 1.15 times (0.986–1.344 95% CI), together with age, tHcy, LDL-C and hypertension.

**Conclusions:**

Circulating resistin levels were comparable in obese/overweight T2DM and control women. In T2DM women, resistin levels correlated with markers of renal function, systemic inflammation and endothelial dysfunction and were independently associated with a higher CHD risk.

**Supplementary Information:**

The online version contains supplementary material available at 10.1186/s13098-021-00715-7.

## Background

Type 2 diabetes mellitus (T2DM) is a chronic and progressive disease characterized by insulin resistance and different degrees of beta cell dysfunction [1]. Cardiovascular disease (CVD) is the leading cause of death in T2DM subjects, especially in women, who lose the estrogenic protection and are exposed to the risk of major CVD events even at younger ages [2, 3].

The pathophysiological basis of the excessive CVD risk in T2DM women is still partly unknown, although several lines of evidence point to a different burden of classical and non-classical CVD risk factors in the two genders [4–7].

Among these, obesity has been demonstrated to confer a higher CVD risk in women than in men with T2DM [8–10].

In addition to its role in energy storage and metabolic balance, adipose tissue is an active endocrine organ releasing several factors involved in systemic inflammation, insulin-resistance and CVD risk, including resistin [11, 12].

Resistin is an adipokine discovered in 2001 as a thiazolidinedione-downregulated gene in mouse adipocytes [13]. In rodents, circulating levels of resistin are increased in obesity, and have been demonstrated to play a role in mediating hepatic and skeletal muscle insulin resistance and in the regulation of glucose metabolism through AMP activation [14–19].

While its role seems to be well defined in animal models, there is considerable controversy on the pathophysiological role of resistin in humans.

Indeed, blood circulating levels of resistin have been shown to be increased in subjects with insulin resistance, T2DM, metabolic syndrome, hypertension and overt CVD [20–23], while other Authors failed to show any significant relationship [24–27].

Furthermore, experimental studies have pointed to the role of resistin in the inflammatory process: the expression of resistin in human peripheral-blood mononuclear cells is upregulated by the proinflammatory cytokines tumor necrosis factor-α (TNF-α) and interleukin-6 (IL-6), and resistin induced the expression of these molecules in white adipose tissue and in peripheral-blood mononuclear cells [13, 28].

The divergent results reported in literature on the role of resistin in human pathophysiology may depend on several factors, spanning from biological aspects to the specific study design and studied population characteristics, also including potential sex/gender-differences. As for obesity [8–10, 29], a different impact of several risk factors in micro- and macrovascular complications has been reported in T2DM men and women [30, 31], and gender-related differences in the association of plasma levels of resistin with anthropometric parameters were also observed [32].

### Aim

Because of the high obesity-related CHD risk in T2DM women, the aim of this study was to explore the relationship between resistin levels and major risk factors, including inflammatory and endothelial dysfunction markers, in overweight/obese T2DM, with and without established CHD, and in a group of women without T2DM.

## Methods

### Study population

A group of T2DM women with (n = 36; 27%) and without (n = 96, 73%) documented coronary heart disease (CHD) and a group of non-diabetic women (n = 55) were consecutively recruited at their first visit among those attending the metabolic disease outpatient clinic of Messina University Hospital and from voluntary employees of the same Institution. Inclusion criteria for both diabetic and control women were a BMI value ≥ 25 kg/m^2^ and being post-menopausal. Menopausal status was defined as amenorrhea for at least 12 months before the study entry or hysterectomy, considering the date of surgery as the beginning of menopause. T2DM was diagnosed according to ADA criteria [33]. All T2DM subjects were on dietary therapy, oral hypoglycemic agents, insulin or a combination between them.

Exclusion criteria for all participants were pregnancy, current treatment with glucosorticoids, inflammatory drugs and thiazolidinediones, hormonal replacement therapy, oral contraceptive use or multivitamin supplementation; cancer or any major medical condition in the last 6 months preceding the study.

Hypertension was defined according to current guidelines [34].

All the participants gave their informed consent and the local ethical committee approved the study.

### Measurements of laboratory parameters

At the enrollment visit, all participants underwent a clinical questionnaire, a complete physical examination and fasting blood sampling for the measurement of study parameters. Anthropometric parameters and blood pressure were measured according to standard procedures.

After a 12–14-h fasting, blood samples were collected from all participants for the determination of the study parameters. Blood was drown in a 10-ml tube containing EDTA (0.15% final concentration) and in a regular 10-ml tube. After collection, plasma and serum were immediately separated at 2500 rpm for 30 min at 4 °C, and aliquots were stored at − 80 °C until analysis.

Fasting plasma glucose and serum creatinine levels were measured with standard automated laboratory methods (Roche Diagnostics, Milan, Italy). Glycated haemoglobin (HbA1c) was measured using an automated high-performance liquid chromatography (HPLC) analyzer (Diamat; Bio-Rad Laboratories, Milan, Italy); normal range values in our laboratory are 4–6%. Fasting insulin concentration was measured by radioimmunoassay (Diagnostic Corporation, LA, CA). Insulin resistance was calculated by the homeostasis model assessment (HOMA-IR) [16 ref].

Plasma total cholesterol and triglycerides levels were measured by automated enzymatic assays. HDL-C was measured directly with a kit from Roche Diagnostics (Indianapolis, IN). Plasma total cholesterol and triglycerides levels were measured by automated enzymatic assays [ref]. Direct low-density lipoprotein cholesterol (LDL-C) was measured with reagents from Equal Diagnostics (Exton, PA). HDL cholesterol (HDL-C) was measured directly with a kit from Roche Diagnostics (Indianapolis, IN).

Glomerular filtration rate (GFR) was estimated by using CKD EPI equation [35].

### Measurements of Resistin, inflammatory and endothelial dysfunction markers

Serum levels of resistin, IL-6 and VCAM-1 were determined by ELISA (R&D Systems, Minneapolis, Minnesota); hsCRP were assayed with a high-sensitivity test (Dade Behring Inc., Deerfield, Illinois); tHcy plasma concentration was measured with HPLC technique (Bio- Rad Laboratories, Milan, Italy; CV 2.9%).

### Assessment of T2DM micro- and macrovascular complications

Diabetic micro-and macroangiopathy were screened according to national and international diabetes guidelines [34, 36].

Diabetic retinopathy was diagnosed based on direct ophthalmoscopy (through a dilated pupil) performed by an expert ophthalmologist and/or by fluorescein angiography within 1 year before the start of study.

Diabetic kidney disease was diagnosed according to estimated Glomerular Filtration Rate (eGFR estimated by CKD EPI) and albuminuria measurements, as the presence of impaired eGFR < 60 ml/min/1.73 m^2^ and/or albuminuria ≥ 30 mg/die.

Coronary heart disease (CHD) was defined as a history of myocardial infarction, chronic ischemic heart disease, coronary heart by-pass, coronary angioplasty, documented by cardiologist medical records and/or hospital discharge. Asymptomatic myocardial infarction or arrhythmia were excluded on the basis of a standard electrocardiogram and cardiologist visit, performed annually in all T2DM patients as part of the usual screening program.

Cerebrovascular disease or peripheral arterial disease were assessed by color-Doppler ultrasonography by B-mode real-time ultrasound, as part of the periodic screening of macrovascular complications.

### Statistical analyses

Statistical analysis was performed using the SPSS program, version 11.0 for Windows (SPSS Inc. Chicago, IL). Data are expressed as mean ± SD; most of examined variables were normally distributed as verified by Kolmogorov–Smirnov test; consequently, the parametric approach has been used. We used χ^2^ test to compare categorical measures, and the analysis of variance (ANOVA) for continuous measures. For each parameter, we performed statistical comparisons between groups applying Student’s *t*-test.

Linear regression models were determined using a stepwise selection procedure in order to assess the possible dependence of resistin on study variables; firstly, we estimated all univariate models; subsequently, a stepwise multivariate regression analysis was performed. All statistical comparisons are two-tailed; a value of *P* < 0.05 was considered to be statistically significant.

## Results

### Clinical characteristics of T2DM and control women participating to the study

Demographic and clinical characteristics of the overweight/obese postmenopausal women (132 T2DM women and 55 controls women; mean age 59 years and 57 years, respectively; P = 0.265) participating to the study are shown in Table 1.

**Table 1:** Clinical characteristics of T2DM and control women participating to the study

	T2DM women	Control women	P
n	132	55	
Age (years)	59.04 ± 12.61	57.0 ± 7.45	0.265
BMI (Kg/m^2^)	31.74 ± 5.94	28.38 ± 4.52	< 0.001
Waist circumference (cm)	100.03 ± 11.61	90.73 ± 14.87	0.0006
Smokers n (%)	17 (12.9)	6 (10.9)	0.29
Systolic blood pressure (mmHg)	134.39 ± 17.35	125.46 ± 12.75	0.0008
Diastolic blood pressure (mmHg)	78.52 ± 9.09	73.15 ± 9.38	0.0003
Diabetes duration (years)	7.77 ± 8.97	–	
HbA1c (%)	7.44 ± 1.44	–	
Fasting plasma glucose (mg/dl)	161.99 ± 50.03	96.75 ± 5.98	< 0.001
Insulin (mU/L)	16.38 ± 10.80	13.82 ± 10.11	0.68
HOMA-IR	6.86 ± 5.72	3.32 ± 2.46	0.0007
Creatinine (mg/dl)	0.89 ± 0.20	0.85 ± 0.10	0.114
eGFR (ml/min/1.73 m^2^)	68.27 ± 14.71	70.66 ± 10.32	0.273
Total cholesterol (mg/dl)	186.74 ± 29.64	201.00 ± 28.26	0.0016
HDL-cholesterol (mg/dl)	48.19 ± 13.69	53.76 ± 12.43	0.010
Triglycerides (mg/dl)	121.99 ± 59.92	99.78 ± 52.86	0.0341
LDL-cholesterol (mg/dl)	113.41 ± 26.50	127.29 ± 28.28	0.0032
LDL-C/HDL-C	2.70 ± 2.26	2.50 ± 0.84	0.522
Hypertension n (%)	87 (65.9)	18 (32.7)	< 0.001
CHD n (%)	36 (27.3)	0	–
Stroke/TIA	0	–	–
Carotid atherosclerosis (%)	34 (25.7)	–	–
Lower limb atherosclerosis (%)	24 (18.2)	–	–
Diabetic nephropathy n (%)	35 (26.5)	–	–
Diabetic retinopathy n (%)	30 (22.7)	–	–
Diabetic neuropathy n (%)	9 (6.8)	–	–
Insulin treatment n (%)	11 (8.3)	–	–
Oral Hypoglycaemic drugs n (%)	109 (82.6)	–	–

Data are n, %, means ± DS

eGFR: estimated glomerular filtration rate

HOMA-IR: homeostatic model assessment

Diabetic nephropathy:EGFR < 60 ml/min and/or albuminuria

T2DM women (mean duration 7.7 years; HbA1c 7.44%), showed higher values of BMI, waist circumference, triglycerides, systolic and diastolic blood pressure, and an higher degree of insulin-resistance as evaluated by HOMA-IR and hypertension rate when compared to controls (P < 0.01 for all comparisons) (Table 1). Conversely, control women showed higher levels of total- and HDL-cholesterol (P = 0.0016 and P = 0.010, respectively). Overall, renal function was acceptable and comparable between the two groups.

Among T2DM women, 27% had established CHD, 22.7% had retinopathy, 6.8% neuropathy, 26.5% nephropathy (eGFR < 60 ml/min and/ or albuminuria > 30 mg/24 h), 25.7% carotid atherosclerosis and 18.2% lower limb disease. Overall, 82.6% were on oral agents and 8.3% on insulin therapy with or without oral agents (Table 1). None of controls had a history of cardiovascular disease or diabetes complications.

Circulating levels of adipokines and inflammatory markers in control, T2DM women and T2DM women with CHD are shown in Table 2. Resistin, IL-6, sVCAM, tHcy as well as folate and vitamin B12 serum levels were comparable between the T2DM and control groups, whereas hsPCR levels were ~ 2 times higher in T2DM than in non T2DM women (P = 0.0132).

**Table 2:** Resistin levels and inflammatory and endothelial markers in T2DM and control women participating to the study

	Control women	All T2DM women	T2DM women without CHD	T2DM women with CHD	P1	P2	*Age- adjusted ****P2***
n	55	132	96	36			
Resistin(ng/ml)	10.31 ± 4.28	10.27 ± 4.11	9.50 ± 3.33	12.43 ± 5.21	0.953	< 0.001	0.013
hsPCR (mg/L)	2.97 ± 3.27	5.54 ± 6.42	5.83 ± 7.34	4.90 ± 4.55	0.0132	0.452	0.682
IL-6 (pg/ml)	2.17 ± 2.07	2.87 ± 2.07	2.72 ± 3.84	3.35 ± 2.03	0.109	0.317	0.913
sVCAM1 (ng/ml)	715.07 ± 202.63	768.65 ± 313.48	707.32 ± 194.80	940.11 ± 481.97	0.245	< 0.001	0.118
tHcy (μmol/L)	11.47 ± 4.58	12.77 ± 5.94	11.68 ± 5.01	15.92 ± 7.25	0.165	< 0.001	0.799

Data are n, means ± DS

P1: P value for the comparison between T2DM and control women participating to the study

P 2: P value for the comparison between T2DM women with and without CHD

Among T2DM women, we did not find any significant difference in resistin levels according to the use of secretagogues drugs (9.85 ± 3.49 vs 10.79 ± 4.75 ng/ml in secretagogues users and non users, respectively; P = 0.191); similarly no differences were noted according to the use of other hypoglycaemic drugs including metformin and/or insulin (data not shown).

Resistin, tHcy and sVCAM levels were significantly higher in T2DM women with CHD compared to those without (P < 0.001, all), and this difference was still significant for resistin after age-adjustment (Table 2).

T2DM women with CHD were older (P < 0.001), with a longer diabetes duration (P = 0.002) when compared to T2DM women without established CHD (Additional file 1: Table S1), whereas the degree of adiposity (BMI and waist circumference values), HbA1c values and lipid profile were comparable between the two groups. Micro- and macrovascular complications, and subjects on insulin treatment were also more frequent in the CHD group than in the non-CHD group (P < 0.05 and P < 0.001, respectively; Additional file 1: Table S1).

### Univariate and multivariate associations of resistin levels with study parameters

**Fig. 1:**
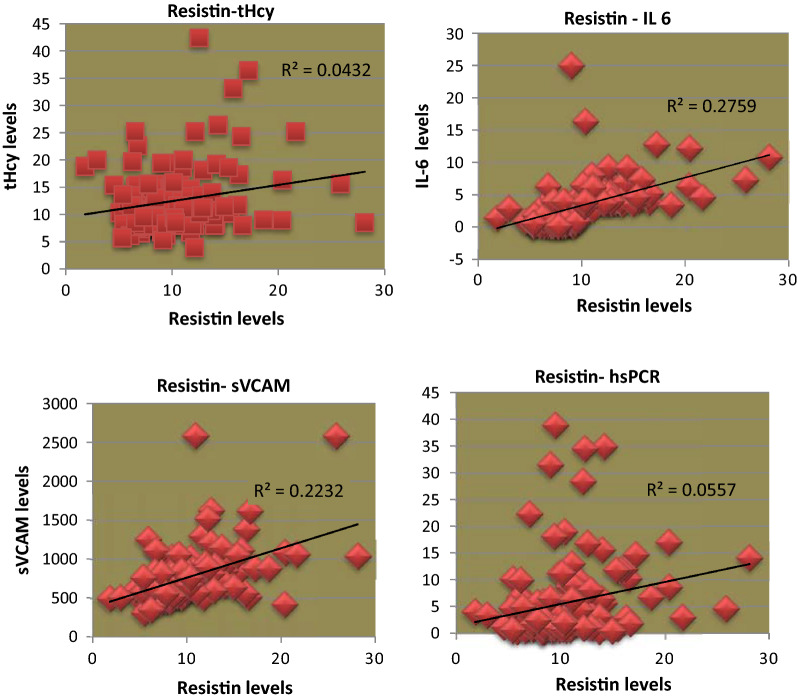
Correlations of resistin levels with inflammatory and endothelial dysfunction markers in T2DM women

Significant correlations were noted between resistin levels and all the investigated inflammatory and endothelial dysfunction markers, although the strength of the association was greater for IL-6 and sVCAM (Fig. 1).

Univariate regression analysis of resistin levels with study variables in T2DM is shown in Table 3. Overall, resistin levels were positively associated with age, hypertension, waist circumference, insulin levels and the degree of insulin resistance (HOMA_IR_), triglycerides and creatinine levels, and inversely with HDL-C concentrations (Table 3). Creatinine and hypertension showed the strongest association with resistin levels. Significant univariate associations were also noted with tHcy, IL-6, hsCRP and sVCAM (Table 3).

**Table 3:** Factors independently associated with Resistin levels in T2D women

	Univariate regression analysis			Multivariate regression analysis		
	OR	95% CI	P	OR	95% CI	P
Age	0.060	0.004–0.116	0.035			
Diabetes duration	0.026	− 0.062 to0.113	0.563			
Hypertension	1.851	0.389 to 3.314	0.013			
BMI	0.097	− 0.016 to 0.211	0.093			
Waist circumference	0.076	0.015–0.136	0.015			
Systolic blood pressure	0.038	− 0.002 to 0.079	0.065			
Diastolic blood pressure	0.055	− 0.024 to 0.133	0.171			
HbA1c	0.119	− 0.394 to 0.632	0.648			
Fasting glucose	0.007	− 0.007 to 0.021	0.347			
Insulin levels	0.071	0.006–0.137	0.033			
HOMA-Index	0.117	− 0.006 to 0.240	0.063			
Creatinine	6.124	2.729–9.518	0.001	5.95	1.888–10.013	0.005
tHcy	0.162	0.042–0.281	0.009			
Total cholesterol	− 0.020	− 0.044 to 0.004	0.099			
HDL-cholesterol	− 0.055	− 0.106 to 0.004	0.036			
LDL-cholesterol	− 0.022	− 0.046 to 0.003	0.079			
Triglycerides	0.012	0.002–0.22	0.025	0.02	0.006–0.034	0.005
hsCRP	0.135	0.039–0.232	0.006	0.152	0.047–0.258	0.005
sVCAM	0.005	0.003–0.007	< 0.001	0.003	0.001–0.006	0.009
IL-6	0.391	0.150–0.632	0.002	0.322	-0.085–0.729	0.045

At multivariate analysis, creatinine, TG, hsCRP, IL-6, and sVCAM were independently associated with resistin concentrations in T2DM women.

### Factors associated with CHD in T2DM women

At stepwise regression analysis, resistin levels were independently associated with CHD, after taking into account a large set of potential confounders, including age, renal function, BMI, waist circumference, glucose control, blood pressure, lipid profile, inflammatory and endothelial dysfunction markers. Age, tHcy, LDL-C levels and hypertension were also independently associated with the occurrence of CHD in T2DM women participating to the study (Additional file 1: Table S2).

## Discussion

We investigated resistin concentration and its potential associations with CVD risk factors, as well as with inflammation and endothelial dysfunction markers in a group of outpatient obese post-menopausal women.

We found that circulating levels of resistin were similar in T2DM and non-diabetic women, whereas they were significantly higher in T2DM women with CHD.

CHD is a multifactorial disease, but the relative impact of risk factors may differ according to gender [5, 6, 30, 37].

Among these factors, obesity has been demonstrated to confer a higher cardiovascular and mortality risk in T2DM women than in men [6, 9, 10]. Adipose tissue is an active endocrine organ releasing several adipokines and inflammatory factors, including adiponectin, leptin, resistin, chemotactic protein 1 (MCP-1), TNF-α, IL-6, IL-1β, IL-10, and transforming growth factor (TGF)-β, which may contribute to the differential impact of obesity in the two genders.

Thus, serum leptin levels correlates positively with body fat content in either sex, but its levels are two times higher and its secretion rate is one third higher in women than in men [38]. Similarly, also hsCRP levels are usually higher in women than in men, whereas men have higher levels of IL-6, Il-8, and TNF-α [39].

To date, data on potential gender differences in resistin levels, and specifically in T2DM women are sparse. It has been reported that the association between resistin and obesity is stronger in women than in men [40], although opposite results were reported in other studies [41]. Thus, a recent study on 92 T2DM subjects reported no significant difference in resistin levels according to gender and BMI, while gender- and BMI-related differences emerged for leptin, adiponectin and visfatin levels [24].

In our study, women with CHD were older than those without it, but the association of resistin levels with CHD risk remained significant after adjustment for age and other potential major confounders, including renal function.

Post-menopausal women with and without T2DM participating to the study were comparable for renal function, which is an important factor potentially influencing resistin concentrations [42, 43]. Thus, also in our series resistin levels were strongly associated with renal function, and creatinine levels were significant determinant of resistin concentration after multiple adjustment at regression analysis. Accordingly, Moreno et al. reported that renal function strongly influenced resistin levels in a large cohort of T2DM subjects, although the association was stronger in men than in women [44].

Conversely, resistin levels were not associated to BMI in our study. Similar to our data, resistin levels have been reported to be not significantly different according to BMI in a recent study [24]. Similarly, Hansen et al. [25] did not find any difference in resistin level in obese and non-obese T2DM subjects. As in our series, other Authors failed to find any difference in resistin levels according to the presence of T2DM and/or obesity [26, 27, 45], whereas other recent studies only found minor differences [46].

On the contrary, Mabrouk et al. showed significantly higher resistin concentration in obese diabetic *vs.* obese non-diabetic subjects, as well as in obese diabetics and non-diabetics *vs.* slim healthy persons [47].

Several factors, including dysfunctional adipose tissue, may contribute to the observed discrepancies in literature data. Beyond the role of renal function as a major determinant of resistin levels in our as well as in other studies, our hypothesis is that in our cohort, resistin levels are similar in women with and without T2DM, because of a similar degree of dysfunctional obesity, which is not captured by BMI values. In women with CHD, the higher degree of chronic inflammation and endothelial dysfunction is likely linked to the higher resistin levels.

Moreover, our data showed that resistin levels correlated with inflammatory and endothelial dysfunction markers in T2DM women, suggesting that the association of resistin with CHD risk may be mediated by the inflammatory process. A huge amount of literature has established the link of systemic inflammation, T2DM and CVD risk, although these evidences failed to be translated in clinical recommendation so far.

Our data are in accordance with several studies showing that resistin plays a major regulatory role in the inflammatory response [48, 49]. Resistin has also been reported to upregulate the expression of proinflammatory cytokines such as TNF-α, IL-6, IL-12, and monocyte chemoattractant protein in PBMCs, macrophages, and hepatic stellate cells via the nuclear factor-κB pathway [47, 50]; the correlation of resistin levels with inflammatory and fibrinolytic markers has been reported both in the general population and in individuals with T2DM, coronary atherosclerosis, chronic kidney disease, rheumatoid arthritis, and/or sepsis [48, 49, 51].

A recent study conducted in subjects with and without ventricular dysfunction showed resistin levels were overall comparable to that of our study, and correlated with IL-6 and HDL-C/TG, but not with renal function as in our study. Younger mean age of participants [50] may partly explain the observed differences with our study.

The pathophysiological relationships among obesity, insulin resistance and subclinical inflammation are complex and not fully explored yet.

, In our study, resistin levels correlated with the degree of insulin resistance, as assessed by HOMA_IR_, and with all the components of the metabolic syndrome (waist circumference, hypertension, HDL-C/TG levels), although the association remained significant only for TG at multivariate analysis. Chedraui et al. demonstrated that post-menopausal women with metabolic syndrome displayed significantly higher levels of resistin, together with higher leptin and insulin levels, differences mainly observed among women with abdominal obesity, as in our cohort [52]. A recent systematic literature review and meta-analysis, including fifteen studies found that resistin levels were weakly correlated with insulin resistance in T2DM and obesity, but this association was stronger in subjects with hyperresistinemia (≥ 14.8 ng/ml) [53]. In our study, resistin mean values were below that cut-off, and the association with HOMA IR was weak and disappeared at multivariate analysis.

The potential role of hypoglycaemic drugs in modulating resistin concentration is another relevant issue. The few available literature data report that thiazolidinediones and sulphonlyureas may alter resistin levels [54, 55]. Thiazolidinediones users have been excluded from current analysis, as for study design. When we analyzed resistin levels according to the use of secretagogues and other hypoglycaemic drugs, including metformin and insulin, we did not find any significant difference, in obese women with T2DM participating to the study.

Furthermore, significant associations were shown between resistin and tHcy and sVCAM levels, that were all comparable in T2DM women and controls, but increased in T2DM women with CHD. Human resistin may play an important regulatory role in the modulation of the interaction between endothelial cells, monocytes/macrophages, and VSMC in the pathogenesis and progression of atherosclerosis, as demonstrated in several experimental [56, 57], and clinical studies [58].

In our study, resistin was also independently associated with a higher CHD risk in T2DM women, after taking into account a large set of covariates.

A causal- effect relationship cannot be ascertained from our data, and for other inflammatory and not conventional CVD risk factors, it is overall difficult to establish whether these molecules are directly responsible for the atherosclerotic damage or their circulating levels are only markers of the underlining pathophysiological process.

In a cohort of 284 T2D patients (48% females), followed-up for 5.4 years, higher resistin levels were associated with reduced survival, and resistin concentration ≥ 11 ng/ml was an indicator of unfavorable outcomes [59], a cut-off exceeded in CHD women in our cohort. Several other studies reported high levels of resistin in patients with established CVD, including myocardial infarction, recurrent ischemic events, and overall with CVD complications [48, 57]. These data are in line with our results and point to a potential pathophysiological relationship of resistin with CVD risk in obese T2DM women.

Several limitations should be acknowledged when interpreting our results. The first deals with the cross-sectional study design and the relatively small number of subjects included in this analysis. However, several important confounding factors including lipid profile and major inflammatory and endothelial dysfunction markers were considered in our analysis.

The lack of data on genetic variants on human Retn gene, that have been correlated with Retn expression and resistin levels in metabolic disorders, including T2DM and obesity [28] is another potential limitation.

## Conclusions

In summary, our data show that circulating resistin levels are comparable in overweight/obese post-menopause women with and without T2DM, but they are independently associated to CHD in this cohort. Furthermore, resistin levels were associated with renal function, atherogenic lipid profile, and with inflammatory and endothelial dysfunction factors.

There is an urgent need to define gender-specific CVD risk factors in T2DM, and our data add a piece of information on the debated role of resistin in this field.

## Supplementary Information

**Additional file 1: Table S1.** Clinical characteristics in T2DM women with and without established coronary artery disease (CHD). **Table S2.** Factors independently associated with established coronary artery disease in T2DM women participating to the study.

## Data Availability

The data that support the findings of this study are available from the corresponding author, upon reasonable request.

## Abbreviations

T2DM: Type 2 diabetes mellitus
CHD: Coronary heart disease
sVCAM: Soluble vascular cell adhesion molecule-1
tHcy: Homocysteine
hsPCR: High-sensitivity C-reactive protein
CVD: Cardiovascular disease
