# Supplementary material for: Resistin levels and inflammatory and endothelial dysfunction markers in obese postmenopausal women with type 2 diabetes mellitus
Source: Diabetol Metab Syndr. 2021 Sep 8;13:98. doi: 10.1186/s13098-021-00715-7 (PMC8427860; doi:10.1186/s13098-021-00715-7)
Supplement: Supplementary file 1 — Additional file 1: Table S1. Clinical characteristics in T2DM women with and without established coronary artery disease (CHD). Table S2. Factors independently associated with established coronary artery disease in T2DM women participating to the study. [file 13098_2021_715_MOESM1_ESM.docx]

### Table S1 - Clinical characteristics in T2DM women with and without *established* coronary artery disease (CHD)

|  | T2DM women  without CHD | T2DM women  with **CHD** | P |
| --- | --- | --- | --- |
| n | 96 (72.73) | 36 (27.27) |  |
| Age (years) | 54.94±11.46 | 69.97±8.36 | **<0.001** |
| Diabetes duration (years) | 6.19 ±8.38 | 12.26±8.70 | **0.002** |
| BMI (kg/m^2^) | 32.01±6.59 | 30.27±4.87 | 0.151 |
| Waist circumference (cm) | 99.95±11.97 | 100.31±10.66 | 0.887 |
| Hypertension n (%) | 54 (56.2) | 33 (91.7) | **<0.001** |
| Smokers n (%) | 17 (17.7) | 0 |  |
| Fasting plasma glucose (mg/dl) | 161.28±48.99 | 163.78±53.44 | 0.799 |
| Insulin (mU/L) | 15.88±10.81 | 17.71±10.47 | 0.385 |
| HOMA-IR | 6.48±5.35 | 7.90±6.58 | 0.205 |
| HbA1c (%) | 7.44±1.46 | 7.46±1.42 | 0.943 |
| Total cholesterol (mg/dl) | 188.71±30.28 | 178.78±26.92 | 0.086 |
| HDL-cholesterol (mg/dl) | 48.31±13.80 | 47.90±13.55 | 0.879 |
| Triglycerides (mg/dl) | 116.92±72.53 | 135.53±56.97 | 0.168 |
| LDL-cholesterol (mg/dl) | 120.95 ± 28.26 | 113.05 ±24.83 | 0.148 |
| Creatinine (mg/dl) | 0.85±0.13 | 1.01±0.30 | **<0.001** |
| eGFR (ml/min/1.73 m^2^) | 72.00±13.05 | 58.35±14.44 | **<0.001** |
| *stroke/*TIA | 0 | 0 | **-** |
| Carotid atherosclerosis n (%) | 20 (20.8) | 14 (38.9) | **<0.001** |
| Lower limb atherosclerosis n (%) | 17 (17.7) | 7 (19.4) | 0.086 |
| Diabetic retinopathy n (%) | 17 (17.7) | 13 (36.1) | **<0.001** |
| Diabetic neuropathy n (%) | 4 (4.2) | 5 (13.9) | **0.015** |
| Diabetic nephropathy n (%) | 21 (21.9%) | 14 (38.9) | **<0.001** |
| Insulin treatment n (%) | 3 (3.12) | 8 (22.2) | **0.001** |
| Oral Hypoglycaemic drugs n (%) | 82 (85.4) | 27 (75) | **<0.001** |

Data are n, %,means ± DS .

### Table S2 - Factors independently associated with establishe*d* coronary artery disease in T2DM women participating to the study

|  | **B** | **OR (95% CI)** | **P** |
| --- | --- | --- | --- |
| Age | 0.144 | 1.154 (1.069-1.247) | <0.001 |
| LDL-Cholesterol | 0.026 | 0.978 (0.947-1.004) | 0.055 |
| Hypertension | 0.201 | 1.554 (1.464-1.648) | <0.001 |
| Resistin levels | 0.141 | 1.151 (0.986-1.344) | 0.037 |
| tHcy levels | 0.193 | 1.213 (1.026-1.434) | 0.024 |
